# A Report on Epidemics and Endemics

**Published:** 1861-05

**Authors:** O. C. Gibbs

**Affiliations:** Frewsburg, New York


ART. II.—*A Report on Epidemics and Endemics.* Read before the Medical Society of Southwestern New York. By O. C. GIBBS, M.D., of Frewsburg, New York.

CHOLERA.—The epidemic to which attention is next invited, in the furtherance of this report, is cholera. This disease is probably more recent in its origin than the yellow fever or the plague; but, while its history and origin may not be obscured by the mists of ages, its unfortunate victims are already numbered by millions. Its onset is not less sudden, its attacks not less fatal, and the field of its ravages is far less circumscribed than that which characterizes the two epidemics already alluded to.

Perhaps the first well-authenticated visitation of this disease, as an epidemic, was in 1762, when, in the low and damp regions bordering on the Bay of Bengal, it prevailed with a terrible malignancy, destroying many thousand lives. In 1780 and 1781 it again prevailed in the same region, and perhaps with a still greater mortality than in 1762. Its attacks were sudden and dreadfully fatal, killing mostly within the first hour: it is said that thousands dropped dead amid the mirth and rejoicing of festivals.

It was not, however, until the year 1817 that the attention of the physicians of Europe and this country was prominently directed to the ravages and spread of this disease. In that year it attacked the Indian army, organized for a crusade against the Hindostan chiefs, and in twelve days it is said that more than 10,000 perished. The carnage of battle and the ravages of famine were nothing in comparison with the dreaded and deadly visitations of the unseen pestilence. Jessore and Calcutta were dreadfully desolated. From Calcutta it spread westward along the valley of the Ganges to the interior of Hindostan; southward along the Coromandel coast to Madras, and eastward into the Birman empire. In 1818, 1819, and 1820, the disease slowly spread in the directions indicated, along the valleys of streams and by the sea-coast. It was not until the year 1821 that it reached the shores of the Persian Gulf, and did not fairly enter Europe until 1830.

Two years subsequently it made its first appearance in this country at Quebec, and only sixteen days later at New Orleans. From Quebec it spread westward along the St. Lawrence and the shores of the Lakes to the valley of the Mississippi. From New Orleans it extended northward along the Atlantic coast and up the valley of the Mississippi, westward through Texas and Mexico to California, south and eastward to Vera Cruz and the West India Islands. From 1834 to 1846 the disease was confined principally to the jungles of the Gangetic delta. Early in the summer of the last-mentioned year the disease aroused itself into unwonted activity and spread with great rapidity over nearly the whole field traversed during the former epidemic, which commenced in 1817 and ended in 1834. It reached this country in December, 1848, and in 1849 it progressed northward, reaching Chicago in April, New York in May, Buffalo in June, and Sandusky in July. Nearly all the intervening towns on the Atlantic coast, and on the banks of the Mississippi River and some of its larger tributaries, were more or less severely visited. The disease was epidemic in the West India Islands in September, and in California in October of the following year. In 1853 it was again epidemic in Europe, and in 1854 in this country, since which time, in the United States, only isolated manifestations of the disease have occurred.

But few, who have not given this disease special study, have any idea of the great mortality which it has occasioned. Without wishing to recapitulate, it may not be amiss to introduce the following statistics of mortality, occurring in a few of the many cholera visitations, for which statistics I am mostly indebted to the labors and researches of others.

During seventeen years immediately succeeding 1817, cholera did not cease to prevail. Of the mortality of the first two or three years no reliable statistics are at hand. In the year 1819 more than 10,000 perished of the disease in the Island of Ceylon. In 1820 it raged terribly in the Birman empire; at Bankok alone 40,000 perished; deaths occurred with such rapidity that it was impossible for the living to bury the dead. In the same year the deaths from cholera in the Island of Java were estimated at 400,000; and at Muscat, on the western coast of the Gulf of Ormis, in 1821, 10,000 perished in ten days. About the same time at Bassora, on the north of the Persian Gulf, 18,000 died in two weeks; and in the vicinity of Shiraz 40,000 fell victims to the disease. In 1823 the cholera raged so terribly in Pekin and Nankin that government authority had to superintend the burials and meet the expenses of interment.

In 1830 the disease was epidemic at Moscow, and 3000 fell victims to it. In 1831 18,000 perished of the disease in Paris. In the same year it destroyed 45,000 of the pilgrims at Mecca, and in Hungary its victims were estimated at 18,000. In 1832 more than 30,000 perished with cholera in the British Islands, and in the same year it was very fatal in this country. In 1848 the deaths from cholera in Great Britain were more than 70,000, and in Russia in the neighborhood of 500,000! In 1849 nearly 4000 died of the disease in New Orleans, and nearly 5000 at St. Louis; in 1850 the latter city suffered even more severely. At Cincinnati, in 1849 and 1850, the mortality from this disease was nearly 6000. In 1849 it occasioned about 400 deaths in the comparatively small town of Sandusky, and in 1850 more than 40,000 fell victims to it on the Island of Jamaica.

These figures, great as they are, give but an imperfect idea of the great mortality which this disease has occasioned. It has been estimated that within the last hundred years, and principally within the last fifty, cholera has occasioned full one hundred millions of deaths!—a number not quite equal to about four times the present entire population of the United States! When we take into account this great mortality; when we consider the fact that the disease has probably become naturalized in the delta of the Mississippi, and is probably destined to new incursions and an increased mortality, anything concerning its history, nature, origin, and cause becomes of paramount importance. It is highly probable that the present year will not pass without witnessing a new incursion: how wide spread and fatal cannot be foretold, but the gloomy records of the past justify the most fearful forebodings. The cholera epidemic is again on its western march, and, probably, ere another winter, it will have reaped its harvest of death in this country.*

Are the manifestations of this disease an inscrutable dispensation of Providence, or may we, if we will, see the silent and expressive finger of the All-wise pointing to an improved sanitary and social condition? Is cholera a necessity of our climate, and of man's social and commercial relations, or is it a preventable difficulty, engrafted upon man's folly, and preventable by his wisdom? These are certainly pertinent inquiries, apologetic at least of my humble efforts toward their solution.

The geographical limitations and isothermal relations of cholera are by no means well defined. Unlike yellow fever and the plague, it may occur in high latitudes, and at an undefined elevation. Prof. Dickson has graphically said: "The malignant Asiatic cholera, like the prophet's cloud, 'at first larger than a man's hand,' has covered with its dark shadow almost the whole of the habitable globe, and swept off millions into the tomb. Arising in Bengal, it extended itself slowly over Eastern India, invaded the vast empire of Russia, striking many parts of Germany, and adding the last bitter drop to the miseries of unhappy Poland. Nor seas, nor deserts, availed to arrest its career. It depopulated the crowded streets of Cairo and Constantinople, and whitened, with the bones of armies of pilgrims, the sandy plains which surround the holy cities of the prophet. England and France, enveloped in all the safeguards that science and art can offer for the protection of man from physical calamity, have enjoyed no exemption, and the waves of the broad Atlantic pre-

---

* Written in 1858. Fortunately the disease was not epidemic to any considerable extent in this country in 1858 or 1859.

sented an insufficient barrier. The sea-coast, and the vast interior of our own beloved country, our lakes, and the banks of our majestic rivers, have scarcely ceased of late to mourn its presence; it often reappears, or lingers upon the southwestern portion of our continent, and our cities are depressed with the terrors of its repeated and deeply dreaded invasion. Its victims were multiplied fearfully at Naples and in Palermo, and its mortality, everywhere awfully great, seemed more frightful than ever at the foot of Etna and of Vesuvius."

Cholera generally prevails in the summer and autumn months, and is usually a disease of warm climates; it has, however, occasionally occurred in the winter, and once as far north as Moscow. Notwithstanding cholera has done its deadly work upon the burning plains of British India, and in the cool regions of Norway—occasionally far up the sides of the mountains, and oftener down in the more fruitful valleys—there are certain atmospheric conditions usually present. Those conditions are *heat, high dew point, and stagnant air*. It is not necessary that the heat should be absolute, but relative. I wish to impress this idea, that high temperature, and dew point, and stagnant atmosphere can relatively exist as well in Moscow as in Ceylon or Liberia. The Arctic explorers tell us that the relative high temperature of the Northern Ocean is not less oppressive to the traveler, surrounded though he may be with snow and icebergs, than is the *ordinary* temperature of the sunburnt tropics. But more of this further on. There is one fact that should be mentioned in this connection; wherever cholera manifests itself during one epidemic, there, all things the same, it may be expected during its next visitation. The reason for this, I shall attempt to show hereafter.

A disease that has destroyed, on an average, a million of lives annually, for the last hundred years, and may, with unopposing circumstances, continue to do so indefinitely, is not without interest to the profession and the public. If it be a disease that originates without discoverable cause; is propagated in a manner calculated to baffle the united wisdom of the world; whose deadly march and gloomy shadow, seas nor mountains can oppose, nor sanitary cordons ward off, and whose icy touch, fatal almost as the dart of Azrael, cannot be paralyzed, then may we sit down and receive in meekness the dread visitant, as a chastening messenger from an offended God. That an occasional epidemic visitation of cholera, is a necessary part of the Divine economy, I cannot reconcile to my idea of the non-existence of an ultimate evil. That it was an irrevocable part of the Creative design, that a voiceless and invisible plague should emerge from the jungles of India and slowly spread from house to house and city to city, through valleys and over mountains, across lakes and over the ocean,—marking its pathway with millions of corpses, and still other millions of frightened and fleeing fugitives, uniting in one common sorrow all nations and conditions of men,—involves an idea so derogatory to God, that no enlightened mind can for a moment give it assent.

Effects are not usually produced without causes, and it is probable that cholera is no exception to the general rule. If one country escapes the disease while another suffers—if Norfolk is almost depopulated while Richmond, in the same State, is free from the disease—if one street or one house escapes, in an affected district, there are reasons for the exemption in the one case, and the affliction in the other, and these exemptive conditions, it is reasonable to suppose, can be made universal. That cholera is comparatively a recent disease, perhaps no one disputes. If it was unknown to the ancients, and existing now, it must exist by a special dispensation of Providence, or else its operative cause must have relation to the changing conditions of man's physical existence. The relation of the earth to the source of heat and light upon which climatic and atmospheric conditions depend, is comparatively unalterable through centuries; hence, a new disease is presumed to have causative relation to the vicissitudes of man's physical and social conditions. It is all-important to know what those conditions are, and whether or no they be removable. The great mass of mankind are too apt to regard an epidemic, though it drape the earth in mourning and fill the land with new-made graves, as a providential visitation, and not as a legitimate sequence of man's folly preventable by his wisdom. Men and nations are not so unwise as not to put forth means adequate to such an end, if they have firm confidence in the legitimacy of the desired result. In 1848, cholera destroyed perhaps a hundred thousand of British subjects, fifteen thousand of which were taken from the very capital. If Russia should scatter assassins throughout the British provinces, and in one night 100,000 loyal Britons should fall by the hand of the secret foe, is it not to be supposed that every energy, at whatever cost, would be put in requisition to prevent the repetition of the outrage? Be assured that if the British nation had the same confidence in its ability to stay or prevent the ravages of cholera, efforts commensurate with the design would be put in requisition. In our own country too, cholera and yellow fever have destroyed far more lives, perhaps in one year, than have fallen by the hands of a foreign foe since the day we first aspired to an independent national existence.

Having made the above remarks, I now invite the attention of the Society to the conditions upon the presence of which the existence of cholera usually depends, hoping that causative conditions may be discovered and the possibility of their removal be demonstrated. It has been stated that cholera has been developed under all conditions of climate, temperature, elevation, and atmospheric salubriousness. Apparently this is true, but, absolutely at least, the last clause requires confirmation. One thing cannot be denied, though there may be exceptional developments, the home of the disease is in warm climates, in low situations, and upon unhealthy sites. Reference has been made to the localities where the plague and yellow fever make their attacks and repeat their dreaded visitations, and local causes have been sought and found for these periodic onslaughts upon human life and human hopes. He who observes closely will find that cholera has similar local predilections, feeds upon similar elements, and, when its mission is accomplished, retires to similar winter quarters. In the same cities, upon the eastern continent, where plague and yellow fever have brought sorrow to almost every home, there cholera has done its deadliest work. In the New World reference has been made to Havana, Vera Cruz, New Orleans, Norfolk, and other cities, and the causes of insalubrity have been briefly pointed out: it is in these same and similar localities that men have had cause to mourn the ravages of cholera. Hence, the inference is just that these great epidemics have causes almost identical, and preventive measures that will prove effectual in regard to one may be expected to do so in regard to all. It is as impossible, in my judgment, for a village to be decimated by cholera or yellow fever that is not surrounded by or does not contain within itself the noxious elements of disease, as it is for an individual to suffer from primary syphilis who had not come in contact with its specific poison.

In my remarks upon yellow fever, it was affirmed that that disease only made its appearance where the air was poisoned by putrid, decaying, and noxious exhalations from cess-pools, sewers, churchyards, etc., and this remark will equally apply to the disease under consideration. Dr. Barton, of New Orleans, has affirmed that, for the production of cholera, two conditions are necessarily present: first, a certain epidemic influence, so operating upon the system as to produce a constitutional predisposition; and, second, the introduction into the system of a morbific, decomposing matter, which had its origin in local conditions. The first is an atmospheric condition, the exact nature of which is at present unknown, and is probably beyond the control of man; the latter is a noxious exhalation, intimately connected with human existence and social habits, and consequently preventable. Neither of these conditions is alone adequate to the production of cholera, and if one be preventable, the result, so far as this disease is concerned, is the same as though both were. What is this second element? What are the local conditions without the presence of which cholera cannot exist? These are questions of the first importance. The discovery of the means by which small-pox could be prevented was a boon to humanity, in comparison with which the most learned disquisition upon the pathology and treatment of that disease would sink into insignificance. So with the disease under consideration—the cause and the prevention of cholera are subjects of vastly more importance than its pathology and treatment.

*First* among the local causes of cholera stands *miasm*—a *poisonous exhalation from decomposing animal and excrementitious matter.* It is well known that Dr. Cullen, long ago, observed that the effluvia from putrid animal substances readily produced diarrhœa, and others have confirmed his observation. Is it surprising then that a certain intensity and modification of the causative condition will produce an intensified and modified result? Not at all. It is a generally received opinion that intermittent, remittent, bilious-remittent, and congestive fevers, have a common cause—the differing results or manifestations being due to varying intensity, or collateral modifications of origin. If animal or excrementitious exhalations will develop diarrhœa, it would require only an intensity of cause, superadded to an epidemic predisposing tendency, to produce cholera. That this epidemic influence is inadequate to this end alone, repeated observation has abundantly demonstrated, and in proof on this point the highest authorities upon this subject could be abundantly quoted. Mr. Simons, in his Fifth Annual Report upon the Sanitary Condition of London, says: "The specific migrating power of cholera, (the epidemic influence,) whatever its nature, has the faculty of infecting districts in a manner detrimental to life, only when their atmosphere is fraught with certain products susceptible, under its influence, of undergoing poisonous transformations. * * Through the unpolluted atmosphere of cleanly districts it migrates silently, without a blow: that which it can kindle into poison, lies not there. To the foul, damp breath of low-lying cities, it comes like a spark to powder. Here is contained that which it can swiftly make destructive—soaked into soil, stagnant in water, griming the pavement, tainting the air—the slow rottenness of unremoved excrement, to which the first contact of this foreign ferment brings the occasion of changing, into new and more deadly combinations." Dr. Johnson, in describing those parts of Port Royal in which the cholera was most fatal, says: "Most of the houses have small court-yards attached to them, which are generally the abode of pigs and goats, and are also invariably the depositories of every species of disgusting filth, such as human ordure, as well as other excrementitious matters, stinking fish-guts and putrid slops; in fact, everything is there to be found, excepting cleanliness or pure water. The stench perceivable in the vicinity of some of these localities is at times intolerable. The few inhabitants that do observe anything like decency (there being no public privies) generally resort to the beach facing the sea in front of the battery, in the vicinity of the church. About this spot the night soil is generally deposited. When the sea breeze blows home, this place is directly to the windward of the town." It would not be difficult to multiply descriptive quotations, confirmatory of the fact that in nearly all places where cholera prevails to any considerable extent, there the atmosphere will be found vitiated with poisonous exhalations. While remarking upon yellow fever, I made a tolerably full statement of the sanitary condition of the more important places in which that disease has raged with deadliest effect. It is in the same localities that cholera has raged with the most wide-spread and disastrous results, and to dilate upon miasmatic causatives would be but to recapitulate what has already been said.

Those who advocate that cholera originates only in a specific poison, spreads uninfluenced by terrene conditions, and is altogether beyond human control, may object that there are so many places where filth abounds and noxious effluvia taint the air, that in many places where cholera prevails it is not difficult to show a vitiated atmosphere to be a coexistent condition; and they may, consequently, ask us for proof of a dependent relation between them of cause and effect. This is a more difficult if not an impossible task. I cite a few facts bearing upon this point. In Spanish Town, in 1850 and 1851, cholera was dreadfully fatal. Of the barrack-rooms, Dr. Milroy says: "Nothing can be worse than the construction of these places; they are literally shut boxes over huge pits of ordure, the fluid parts of which soak into the ground, while the solid matter goes on accumulating." Dr. Milroy adds: "The mortality among the well-conditioned class was very limited; it might be counted by units; *whereas that among the mass of the people was by thousands.*"

In 1853 cholera broke out in a limited district in Dublin. The patients all lived near each other, and, "in their immediate vicinity, there was a very large collection, in a yard, of street manure and night soil, collected by carts from the lanes and alleys of the neighboring parts of the city." The balance of the city remained healthy. Instances might be cited in which the disease has confined its ravages to a single ward or street reputed for its filth and nauseous stenches, while the remainder, and salubrious portions of the city, were exempt from the disease, and this same localization has been repeated again and again at each subsequent visitation. For example, "the earliest case of cholera in Chelsea, in 1848, is said to have been in White Hart Court, and there it continued to exist until the end of the epidemic in 1849. The first case in 1854 was in the same place, perhaps also in the same house, for deaths occurred in the same house in both visitations." A writer in the *British and Foreign Medico-Chirurgical Review* for 1857 reports the results of his own observations, which are so confirmatory of the local origin of the disease, that I am disposed to make a rather lengthy quotation. "During the epidemic of 1854, in a portion of the parishes of Chelsea, and of St. Saviour's, and St. George's, Southwark, exclusive of cases in which the notes made at the time of inspection are not explicit, we personally examined into the sanitary state of the houses occupied by 392 families, in whom deaths from cholera had occurred. Out of 2701 persons, 616 had cholera, besides 871 cases of diarrhœa. Four hundred and fifty of the cholera cases proved fatal. The inquiry extended alike to the dwellings of wealthy residents in good streets, as to those of lodgers in the most overcrowded and filthiest alley. Without devoting more space to the subject than we can afford, it would be impossible to convey the evidence in its fullness as it came before us, and tested as it was in every manner that we could think of, but an analysis of the numbers above given shows that the existence of the products of the peculiar decomposition alluded to were evident to the senses at the time of visits in 213 houses. In nineteen of these there were cess-pools situated below the house itself, or in such close proximity thereto that the soil had percolated through into the subsoil below the dwelling. In 220 cases, open privies were either erected against the main wall of the house, or so near to the back entrance as to allow of the emanations from the soil being observable within doors. In seventy-seven instances branch drains of imperfect construction, having direct communication with the common sewer, passed underneath houses; or a foul, open ditch; the main sewer, or a principal branch, in a ruinous condition, was so near the house as to influence its internal atmosphere. In ninety-two of the houses were untrapped sinks or drains, connected directly with the street sewer, by which the foul sewer exhalations were conveyed to the internal atmosphere. Of the entire number of houses, in only thirty-three were no sources of this atmospheric contamination detected. Not to dilate further upon the precise manner in which the air breathed by the inmates became corrupted with this foul impurity, let it suffice that persons appeared to suffer in proportion to the contamination of the air they breathed by the 'privy odor,' and that immunity from this appeared to secure immunity from cholera."

It is repeatedly urged, in opposition to this view of the origin of cholera, that the disease frequently prevails in localities where no tangible cause for insalubriousness is discoverable. This fact does not necessarily invalidate the argument; for local causes often exist in intensity unsuspected by the superficial observer. Those who have searched closely into this matter have been surprised to find poisonous exhalations, in concentrated form, in houses of the upper classes, and in apparently salubrious portions of a city. Again, a prevailing wind may waft away and so concentrate a poison, as to make it operate somewhat remote from the originating conditions. I know, that in all systematic works that have fallen under my observation, it is asserted that the attacks and spread of cholera are uninfluenced by winds. But numerous observed instances, in opposition to this idea, could easily be quoted. In 1849, cholera broke out in the Baltimore alms-house, and ninety-nine died of the disease. The alms-house is placed in an apparently healthy locality, at a distance from the city. At the north of the alms-house was a waste piece of land, of cess-pool soil, which received the filth from the pig-sty, the draining from the privies, and all the filth of the locality. Dr. Buckland says this place was "one putrid and pestilential mass, capable of generating, under the ardent rays of a midsummer sun, the most poisonous and deadly exhalations." The wind set steadily from the north during the epidemic; when the wind changed, so as to waft the exhalations from this abode of filth in another direction, the epidemic ceased at the alms-house. At St. Louis, in 1849, the disease changed locality at every change of the wind.

In my investigations upon the subject of cholera, I had collected a vast array of facts,—all going to show the intimate connection between poisonous exhalations and cholera manifestations. These facts cannot be used, without protracting this paper greatly beyond the indulgence of this society. The above few, of the many facts at command, have been selected, not because of superior force, but because of brevity of statement.

Of high temperature, high dew point, and lowness of site, as collateral agencies in the development of cholera, much might be said. Their influence is wholly operative in enervating the constitution, and in intensifying the effluvial poison. The remarks upon these points, while speaking of yellow fever, are equally applicable here; and want of space will not justify their repetition.

In what respect does an exhalation that will produce cholora differ from that which will develop ague or the yellow fever? This is a pertinent and interesting question, and should not be wholly ignored in this investigation. Intermittent fever is supposed to have for its cause an exhalation, or miasm, derivable from vegetable decomposition. While remarking upon yellow fever, an effort was made to show that that disease had, for its special cause, an exhalation derivable from the putrefactive process, in which animal decomposition was largely involved. It is the opinion of many close observers, that cholera is the morbific result of an exhalation from excrementitious products. This opinion is forcibly urged by a writer in the *British and Foreign Medico-Chirurgical Review* for January, 1851, and January, 1857. Two or three of the thousand facts that might be urged in support of this idea, will here be introduced. Many years ago, and before the first visitation of cholera in England, at a boarding-school, at Clapham, a foul cess-pool was opened, and its contents spread over the garden adjoining the play-ground. Within two or three days, twenty-two boys were seized with alarming diarrhœa and vomiting, with subsultus of the muscles of the arms, and excessive prostration of strength: two died. This case illustrates forcibly the influence of exhalations from human excrements, at a time when no cholera influence was modifying results. Gaimersheim has been dreadfully visited with cholera. Water is so scarce in the town, that cess-pool water is carefully collected in a pool, and preserved for the extinction of fires. Cholera raged only in the vicinity of this pool: nearly one-half of such residents had the disease; and of those attacked nearly one-half died. In Bethnal Green Parish, London, in a space of 400 yards by 150, in fifteen days, in 1849, 211 deaths occurred from cholera. "Among the principal causes of filth, Dr. Gaven places the accumulation of ordure, which appears never to have been removed from the premises; so that, in process of time, the back-yards, in several localities, have been so raised as to be nearly on a level with what might be termed the first floors of the houses." In the west-end of London, "the dwellings, or rather hovels, in which the people live, are unsurpassed in nastiness by anything known in Ireland. The streets, courts, alleys, and yards are without a drop of clear water; there are no sewers in any part of the locality; and all the drainings of the pig-sties and privies are left on the surface, close to the very doors and windows of the houses, or flow lazily into a large stagnant pond, called the 'Ocean,' which is covered with a filthy slime, and is continually bubbling with putrescent gas." The result is, that, during every epidemic of cholera, this is one of the centres of its desolation. Additional facts, of a forcible character, are abundant, to show the influence of excremental exhalations in the production and localization of cholera; but their quotation is not consistent with the scope of this paper. Suffice it to say that, in many instances, privy vaults and cess-pools have been found to have leaked their nauseous contents into wells supplying water to many families. To such families, in many instances, the disease has been wholly confined, or most alarmingly fatal. In other instances, the outlet of sewers, because of low water, have been exposed, and the wind, when blowing in certain directions, would drive the noxious gases into dwellings, in remote and perhaps healthy parts of the town. In such cases, surprising fatality has occurred, under circumstances, at the time perhaps, entirely inexplicable. Still other cases have occurred, where sewers, being dammed up, have regurgitated the foul contents, which they were designed to conduct away, into the palatial homes of the proud and affluent, thus subjecting them to the disease, as well as the poor and lowly, whose hovels are surrounded with the nidus of the epidemic. I beg leave to conclude this part of my subject with a pertinent quotation from the January number of the

*British and Foreign Medico-Chirurgical Review* for 1851: "There is certainly not a more frequent or more perilous source of mischief, during the prevalence of epidemic disease, than foul and obstructed drains; and it is, unfortunately, the fact, that the construction of these works has hitherto been so very faulty, that in many parts of this metropolis, as well as in every town and city throughout the kingdom, instead of being conduits for the removal of decaying refuse, they are no better than elongated cess-pools, which detain their stagnant contents, and regurgitate their noxious effluvia into the interior of the houses with which they are connected."

To this view of the nature of the miasm which develops cholera, it may be objected, that the disease has occasionally occurred in armies upon the field, as well as among our over-the-land emigrants to California, where fecal exhalations must be quite inconsiderable. It may not be easy to meet this objection satisfactorily; though, to my mind, it has but little force. It is not improbable that many of such epidemic visitations have not been cholera at all. Epidemic diarrhœa or dysentery has undoubtedly sometimes been mistaken for cholera. Exposure to the hot sun by day, in an almost uninhabited region rich in decaying vegetation, and also exposure to the damp, cold, and miasmatic air of an open encampment by night, with deficient or unhealthy nourishment, are conditions well calculated to develop diarrhœa and dysentery in their worst forms. In 1850, cholera was reported to have done deadly work among the California emigrants. Assistant Surgeon Wm. Hammond, Jr., at Fort Kearney, wrote: "The California emigrants, between Forts Leavenworth and Laramie, have suffered a good deal from a disease, called by them cholera, and which, in its somewhat rapid and fatal course, very much resembles that malady; but which was nothing more than an acute form of diarrhœa, brought on by excessive imprudence in diet, and exposure to many hardships on the plains, to which they were entirely unaccustomed at home. I have seen a great many cases among the emigrants, and I do not think there has been any true Asiatic cholera upon the plains this summer." (*Statistical Reports of the Sickness and Mortality in the Army of the United States*, p. 78.)

It is a little unfair to deny the existence of cholera in localities where we cannot find what seems to us a true explanation for its appearance; and, however correct this denial may be, I am indisposed to take advantage of it. There is another explanation that suits my purpose better. It is probable that the exhalations from the skin and lungs do not differ materially, in nature, from the excrements from the bowels; certain is it, that confined air can be rendered as deadly poisonous by such exhalations as the foulest cess-pool in London. In proof, I would again instance the

English prisoners confined for a night in the Black Hole of Calcutta: of the 146, but 23 lived to see the morning! Such effects have the skin and lung exhalations on cholera manifestations, that several close observers have supposed that over-crowding was one of the most effectual of local causes. At a work-house in Taunton, containing 276 inmates, who were crowded into a badly ventilated space, scarcely averaging 80 cubic feet of air to each, in 1848, nearly all were attacked with cholera, and 60 died; while the inhabitants of the town were nearly if not entirely unaffected.

At a certain establishment in London, where 1400 pauper children were kept with an average space of 140 cubic feet for each, when no cholera was in the neighborhood, and it was disputed whether there were any cases in town, 300 were suddenly smitten, and 180 died! "I am convinced," says Dr. Clark, of London, "that wherever large numbers of human beings are congregated together, and who eat, drink, and sleep in the same apartment, as in the case with young and old in work-houses,—there the inmates are most liable to suffer."

At Juggernaut, it is an annual visitant. "The town of Pooree contains 35,000 inhabitants, and the number of pilgrims sometimes amounts to 150,000. The inhabitants are usually quite healthy before the occurrence of the festival, which takes place in June or July; but immediately on the arrival of the pilgrims, and when the lodging-houses are literally crammed with inmates, cholera suddenly breaks out, and in the space of a few days hundreds are cut off by it. This is not an occasional or incidental occurrence, it is an invariable one; and the disease, which has thus been generated, as suddenly disappears on the dispersion of the crowd, a few isolated cases only occurring for two or three days after the departure of the pilgrims." It may be asked, what these facts have to do with the point under consideration? I answer, much. Emigrants traveling in companies by land, and armies on the field, and pilgrims en route to and from the Holy City, sleep compactly in their tents, with a space often less than fifty cubic feet to the individual, and their alvine and urinary evacuations are deposited near at hand upon the surface of the earth, and there reek in the air perhaps of a tropical clime. It has been objected, by at least one high authority, that cholera cannot have a miasmatic origin, because it frequently attacks ships at sea. It will be readily seen that this objection has no force, for that peculiar miasmatic poison which we have been considering can be developed on shipboard as well as on land. Many instances directly in point could be introduced, did space permit and occasion justify. I invite your attention only to the Black Sea fleet. In 1854, cholera broke out among the soldiers on shipboard, and was exclusively confined to screw line-of-battle ships. These, Dr. Milroy says, were in several respects defective; "the amount of space, for both sick and well, is too limited; the ventilation is most imperfect, the air of the sick-bay in particular being vitiated by the faulty arrangements of the water-closets and the offensive effluvia arising from the men's latrine." Mr. Rees, surgeon of the *Britannia*, attributes the mortality on board that vessel to the crowded state of the middle deck, to the want of adequate ventilation, and to the accumulation of the foul discharges from the stomach and bowels of the sick.

I have perhaps wearied your patience upon the terrene relations of cholera, but be assured there are but few subjects of more importance. The immortal Jenner discovered a means by which that loathsome disease, small-pox, could be almost, if not entirely, annihilated. The ravages of small-pox have probably never equaled the devastations of cholera; and yet it is the opinion of many of the more observing physicians, that the latter named disease can, if not so cheaply at least as effectually, be prevented.

Is cholera a contagious disease? This is an important question, and should be investigated with the utmost candor, and with strict regard to facts. Unprofessional persons are prone to believe in its contagiousness, and almost every town that has suffered from cholera has witnessed the inhuman results of such a belief. The homeless have been denied a shelter and a couch amid their fellows—with no soothing hand or affectionate word to minister to their dying agonies. Parents have deserted their children, and children their aged parents; husbands their wives, and wives their stricken husbands; and dearest friends have fled from the house of sorrow and of death. If the disease be not contagious, the world should know and be convinced of it, that such inhumanities should not be repeated at each new attack.

A writer in the *New American Cyclopædia* says: "Physicians even were once divided in their opinions on this point, but probably at the present time very few could be found who would maintain its contagiousness." "The whole history of the progress of cholera for the last forty years shows conclusively that, though endemic and epidemic, *it is not a contagious disease.*" There are other high authorities that express an opposite opinion with equal positiveness. Prof. Dickson, in his *Elements of Medicine*, says: "The proofs of this communicability are abundant." Again, "An attack of cholera can only be procured among subjects laboring under the pestilence." Dr. Dickson also says that there seems to him as much evidence of the contagious character of this malady as there is of measles, scarlet fever, hooping-cough, etc. I might quote many high authorities who are enthusiastic advocates of the contagious nature of cholera, but my space will not justify extended reference—two or three must suffice. Dr. Becker, of Berlin, says: "I am a most decided contagionist, and it is the force of facts which has made me so." The members of the Bombay Board conclude their report by saying: "It appears to us incontrovertible, that this disease is capable of being transported from one place to another, as in cases of ordinary contagion or infection, and also to possess the power of propagating itself by the same means that acknowledged contagions do." Dr. Copland, in his *Dictionary of Medicine*, enters into the discussion of this subject with much warmth and at considerable length. I had marked several passages for quotation, but will confine myself to one. He says: "It may be briefly premised that this disease is *never produced* without the presence of a certain leaven, or morbific matter, which, *emanating from the bodies of the affected*, and floating in the air, is respired by those about to be attacked. This is the clear and only inference connected with its transmission that can be deduced from the body of evidence now placed before the reader. Those who argue against its transmissible nature cannot show, since the irruption of the pestilence in India, down to its arrival in this country, and transmission thence to America, a *single instance of its appearance in any place without the previous communication with an infected place or person, of a nature to propagate the malady.*" These are, indeed, high authorities, and their opinions are emphatically stated. It would not be difficult to give opposing opinions from equally high authorities, but opinions amount to but little without the facts upon which they are based, and they are consequently withheld. I have carefully examined all the evidence within my reach, and honestly confess I see no good reasons for considering cholera a contagious disease, in the sense in which small-pox, measles, etc. are contagious. It would give me pleasure to enter into this discussion at length, but this is by no means the appropriate time or place; with simply a thought or two I propose to quit the subject. While upon the subject of yellow fever, I gave a few reasons for considering that disease non-contagious. The arguments there adduced apply with equal force to cholera, and their repetition here would be a useless waste of time. The assertion of Dr. Copland would seem to have weight were it true; but, unfortunately for his dogmatic assertion, it is untrue. Cholera has so repeatedly manifested itself in this country, when it could not be traced to previous communication with affected persons, that space cannot be given here for their repetition.

It may be admitted that cholera has frequently broken out soon after the arrival of vessels from a foreign port with sick on board. But such a fact lacks several requisites to make it conclusive. If cholera broke out at all, it could not well be otherwise than under circumstances as above. The commercial relations between foreign ports and our principal sea-port towns are so brisk, and arrivals so frequent, that any cholera attack at

New York or New Orleans, would necessarily be soon after such an arrival. And, further, at the times of the year when cholera usually makes its first attacks, scarcely a vessel arrives from a foreign port without bad cases of diarrhœa or dysentery on board, that, with people excited with the dread of cholera, would readily be dignified with the name of the latter. There are those who suppose the weekly quarterings of the moon control all the changes in the weather; and whenever a remarkable change takes place, with pride and a fancied evidence of proof, they will refer us to the fact that it was within three days, one way or the other, of such reputed quarterings! Such men forget that the moon's changes are as great one day as another, and our contagionists forget that, in our principal ports, vessels are of frequent arrival. If this argument be objected to as unfair, the following upon this point is of undeniable force. It is not enough for the contagionists to show that cholera frequently breaks out after the arrival of a suspected vessel, but they must show that there was *actual communication* between the sick on shipboard and those attacked on land. Such communication may be inferred, and so all subsequences may be inferred to be consequences, but without positive proof such inferences are valueless. All the sources of information at my command, which, I grant, are limited, I have carefully examined with reference to this point, and must confess I was not disappointed in the result. Probably in nine-tenths of the instances, as above referred to as proof of contagion, no such communication is shown. On the other hand, where cholera patients have been brought to an unaffected town, and cholera has soon after appeared among the residents, in many instances it is not difficult to show that no communication between the sick and the new attacks has been held. In such cases the ignorant suspect contagion, but the observing physician looks for a local cause, confident that the disease would have manifested itself just as soon had no cases been imported. The contagionists tell us that the attacks are largely among those in communication with the disease. Supposing this to be so, it lacks an essential element to constitute proof of their position. The first attacks are where the local causes are most concentrated, and if persons going from a healthy locality to minister to the sick subsequently sicken, it is quite as reasonable to suppose that it is *because they have been exposed to the same local causes,* as to suppose it is because of the communicability of the disease. To take the disease in a poisoned house or infected district, it should be remembered is no proof of communicability from person to person. If a man should take the ague in a locality where the elements necessary for the production of that disease existed in their intensity, and his wife and brother from the Green Mountains, in Vermont, should go to minister to his wants, both, in a little time, might find themselves prostrated with the same disease—other friends might follow with like result, and the disease be pronounced contagious; and yet every physician would pronounce their reasoning false and their conclusions incorrect. Should a man take cholera at Mobile and be immediately removed to an airy farm-house on Spring Hill, in the rear of the town, where no source of noxious exhalations were to be found, and the inmates of his new home should be stricken down, never having been away to suspicious localities, such a case would be troublesome proof of contagion. But, so far as I know, no such case is on record. Small-pox, measles, etc. can be communicated from person to person, anywhere and under any circumstances short of previous protection; not so with cholera or yellow fever. But I must not discuss this subject further.

The above remarks are made in the full consciousness of the responsibility of advocating a doctrine, which if it be an error and acted upon, will cost the sacrifice of many valuable lives. It is only because of my firm conviction of the truth of the local origin of the disease and of the possibility of its prevention, that I urge these opinions. If the disease can only be received by communication with those already suffering, then, in times when the disease is prevailing, an efficient quarantine regulation will save the inmates of a city from danger and death. But if the disease is not contagious, but is only produced by the action of the local cause in conjunction with the epidemic influence, then quarantine regulations are of but little moment. In my judgment, far too much time and money has been wasted on quarantine regulations. If, in New York or Philadelphia, requisite attention is paid to cleanliness—if all the filth is removed and the sources of noxious exhalations suppressed; if cess-pools are cleansed and sewers are kept in working order; if all privy vaults are properly connected with sewers, properly arranged and cleansed; and if all noxious exhalations are corrected by proper disinfectants wherever discovered, there need be no fear of cholera, though no quarantine regulations are observed. But if filth and excrementitious products are allowed to accumulate in cellars, by-streets, and back-yards; if sewers are allowed to become obstructed, and privy vaults and cess-pools to send up their noxious exhalations to poison the hot and stagnant air, when cholera influence is abroad in the land, no quarantine cordon, however efficient, will prevent an irruption of the disease. But can these sanitary regulations be put in requisiton? Yes, and should be. The Registrar-General of London, speaking of the dreadful mortality of Salisbury, says: "Any attempt to improve the general drainage of Salisbury would be impracticable; *it would interfere with too many interests.*" It is my opinion that the Registrar-General has over-rated the selfishness of Salisbury. Any nation that can overbear all interests when a railroad is to be built, or command almost any amount of men or money to repel a foreign foe, can, when she is impressed with the necessity and has confidence in the utility, effect such sanitary regulations as are necessary to save 50,000 of her loyal subjects annually! The public is apathetic on all subjects where the pecuniary benefits are not tangible to the least observing, and it is only by an earnest and unyielding effort on the part of the medical profession that proper sanitary regulations can be enforced.

It was not my intention in this report to remark upon the treatment of any epidemic or endemic in which my personal experience was not sufficiently large to warrant a confident expression. If the cause of the disease is as above stated, it will be readily perceived that sanitary regulations and the free use of disinfectants, as preventive measures, will doubtless be of more service than any treatment. It would also seem reasonable to suppose that the administration of chlorine would form a part of all judicious medication. In this connection it might be observed that Dr. Mann, of Clerkenwell, exhibited chlorine water in nearly one hundred cases with a loss of only two patients.

When this report was commenced, the design was to continue it until the various diseases ranked under the head of epidemics and endemics had been considered. From this stage on, it was designed to make the *treatment*, of the respective diseases considered, an important feature in the report. The ultimatum of the design was a work upon epidemic and endemic diseases, of which the two papers now published were but the initiatory. My design having been interrupted by unforeseen circumstances, and my time now entirely occupied by other duties, I shall be compelled, at least for a time, to postpone its completion.

